# Perceived barriers to maintain physical activity and its association to mental health status of Bangladeshi adults: a quantile regression approach

**DOI:** 10.1038/s41598-023-36299-7

**Published:** 2023-06-02

**Authors:** A. B. M. Nahid Hasan, Azaz Bin Sharif, Ishrat Jahan

**Affiliations:** 1 Department of Public Health, North South University, Dhaka, Bangladesh; 2 Global Health Institute, North South University (NGHI), Dhaka, Bangladesh

**Keywords:** Health care, Medical research, Epidemiology

## Abstract

Insufficient physical activity and unhealthy lifestyle preferences have been significant concerns for decades. This study aimed to determine the perceived barriers to maintaining physical activity among adults in three major cities of Bangladesh and their association with mental health status. This is a cross-sectional study where 400 participants were selected using a multistage sampling technique. Twenty municipal wards were randomly selected from three cities, followed by a convenient selection of the study participants from each ward. Questionnaires about perceived physical activity barriers were developed based on previously published literature. The DASS-21 scale assessed the mental health status of the study participants. Descriptive statistics were applied to narrate the baseline characteristics of the respondents. The Shapiro–Wilk test was used to check the normality of the perceived physical activity scores. Quantile regression analysis was applied to model the physical activity barrier scores depending on several covariates. Five quantiles were used: the 10th, 25th, 50th, 75th, and 90th. A p-value less than 0.05 was considered significant for hypothesis testing. Among the respondents, 68.50% were male, half of them were married, 68.0% belonged to nuclear families, 48.0% completed graduate level education, 34.25% were service holders, one-third of the respondent's working hours were 6–8 h, and 19.50% belongs to the overweight and obese groups. Poor traffic and construction work near the road (60.30%) was seen as the most significant barrier to physical activity. Over half of the respondents stated that lack of time, facilities, and expenses hinder physical activity. Mental health status reported mild to extremely severe levels of depression (32%), anxiety (47%), and stress (42.50%), respectively. Significant associations between the perceived physical activity scores and gender, family type, occupation, income, BMI, anxiety, and depression were obtained. Ensuring a safe environment, facilitating accessibility and availability of low-cost exercise facilities, improving road and traffic conditions, and providing appropriate mental health counseling may help to mitigate physical activity barriers.

## Introduction

Globally, approximately one out of four adults (27.5%) does not fulfill the World Health Organization (WHO) recommendations for Physical Activity (PA)^1,2^. Physical activity participation rates haven't changed much over the last 20 years, and there are significant differences in participation between men and women^2,3^. There are numerous physical and mental health advantages to regular physical activity, such as lowering the risk of physical ailments like heart disease and stroke, obesity, cancer, diabetes, etc^4^. Physical activity, a lifestyle modification behavior, lower the likelihood of all-cause death by around 33%^5^.

The absence or insufficiency of physical activity has numerous adverse health outcomes. About 3.2 million people die every year because they don’t get enough exercise^6^. Physical inactivity is linked with higher odds of heart disease, hypertension, diabetes, cancer, and other non-communicable diseases (NCDs)^7,8^. Approximately 74% of all deaths worldwide are caused by NCDs, which kill 41 million people annually^9^. Deaths from NCDs are anticipated to rise to 52 million in 2030^10^. A recent statistic from WHO revealed that77% of all non-communicable disease deaths occur in low- and middle-income countries (LMCIs)^9^. In Bangladesh, NCDs are the significant cause of mortality, responsible for 67% of total deaths^9^. The prevalence of NCDs in Bangladesh has increased over the last ten years, and it is predicted to grow in the coming years as different epidemics are evolving every now and then^11^.

Numerous factors are believed to influence insufficient physical activity engagement. Physical inactivity results from a combination of barriers and sedentary lifestyle choices^12^. Perceived barriers to physical activity are aspects that an individual perceives as impediments or barriers to physical activity^13^. It can be classified into physical, psychological, environmental, and socio-ecological barriers^14^. A study reported that time constraints, lack of motivation, energy, self-discipline, discomfort, expenses, illness and injury, etc., are the perceived barriers to physical activity^15^. In a large-scale survey, lack of time, motivation, and energy have also been identified as major perceived barriers to physical activity^16^. Uddin et al. conducted research in Bangladesh and found that the most common reasons people didn’t get enough physical activity were bad street lighting at night and lack of a suitable place to exercise. Unlike men, women said that a lack of safety in the neighborhood, bad street lighting, dirty and messy neighborhood, and bad weather kept them from being active^17^.

According to previous research, different types of physical activity may have different health outcomes^1^. Most studies have focused on barriers and facilitators to physical activity rather than exploring the unique factors that influence physical activity behavior in specific domains. Understanding domain-specific barriers and facilitators to physical activity is essential for developing effective interventions and policies to promote physical activity in different settings^18^. The barriers to physical activity may differ depending on the population, culture, and socioeconomic situation of the nation, in addition to how those barriers are perceived in the various domains. For instance, a study conducted in Australia found that among those who were physically inactive, time constraints (50.0%) and a lack of enjoyment (43.9%) were the two most frequently cited barriers^19^. Work and the oppressive heat were the two most commonly reported barriers, according to a separate study conducted in Kuwait^20^. These studies show diverse barriers perceived by people from different regions. In Bangladesh, no studies have looked at perceived barriers to physical activity in four important domains: physical, psychological, environmental, and socioeconomic barriers. Moreover, among the studies in Bangladesh, Sadia et al.^21^ surveyed physical activity barriers only in Diabetic patients, and Uddin et al. emphasized on the environmental barriers among young adults in Dhaka city^17^. Being physically inactive increases the risk of mental health problems, such as depression and dementia, and physical health risks like obesity and chronic conditions^22^. Physical inactivity is an important factor that can cause mild to moderate mental distress, such as depression and anxiety^23^. According to the WHO, adequate physical activity is associated with a lower incidence of mental disorders and is beneficial in treating common diseases^23,24^.

Bangladesh has been one of the role model economies in recent years^25,26^. However, the economic growth was concentrated only in major cities and not decentralized. The capital, Dhaka, the port city Chittagong, and the Gazipur city corporations have all seen an influx of rapid urbanization and industrialization^27^. Many people live in these cities, accounting for roughly one-fourth of Bangladesh's total population^28^. In addition to the population density, which is a problem for the whole country, there are similarities in other characteristics between the selected municipalities and the rest of the country. First, every major city is experiencing urbanization, meaning many industries are being built in major cities that attract migrants from the neighboring rural communities. The consequences are that green spaces are disappearing, number of parks, playgrounds, and other recreational facilities are fast diminishing, fewer people have spear time for activities, etc^29^. Because of a rapid urban-industrial transformation and modernization, Bangladeshi people are more technology-centered now than in the past, with increased access to labor-saving technologies in occupational and domestic settings, increased reliance on motorized transports, and more time spent using screen-based entertainments^30^. Moreover, modern technologies and the availability of personal computers, tablets, mobile phones, and video games make the city population less engaged in physical activity. According to the literature, the status of both physical activity and mental health barriers are quite diverse in developing countries as oppose to the developed countries^31^. The reasons for not doing physical activity in the developed countries are not due to insufficient facilities, which is the case in developing countries^31^. On the contrary, poverty and income are considered prime contributors of mental health problems in developing countries but not in the developed countries^15,17,32^. In a developed country, perceived barriers to physical activity include lack of motivation, lack of time, lack of support, particularly lack of a partner, poor weather (temperature, precipitation, wind), fatigue, and illness^22,33–35^. In addition, mental distress, including depression, anxiety, stress, and fatigue, are considered barriers to physical activity in developed countries^34,36^. In developing countries, perception of barriers to physical activity are lack of free time, feeling tired, pollution in the neighborhood, bad weather, poor economic status, lack of knowledge, and feeling fit^15,17,21^. In response to the rising burdens, a major global health emphasis is required to develop and implement policies that promote a healthy lifestyle and ensure physical activity^37^. Understanding perceived barriers to physical activity and initiating strategies to overcome them may facilitate incorporating physical activity into one's daily routine. Therefore, this study aimed to discover the perceived barriers to maintaining physical activity among adults in Bangladesh and explore the relationship between these barriers and mental health characteristics.

## Results

### Sociodemographic characteristics

Table 1 shows the sociodemographic characteristics of the respondents who were selected from Dhaka city corporations (50.50%), Chattogram city corporations (29.0%), and Gazipur city corporations (20.5%), respectively. Most participants were male (68.50%), and 66.75% were aged between 18 and 30. Half of the sample were married, and around 68.0% of the study population belonged to nuclear families. About 48.0% of participant's educational qualifications were graduation or above. Only 30% of the participants studied biological science in their academia. Although 34.25% of participants were service holders, the rest two-third of respondents were business people and other professionals (homemakers, unemployed, students). One-third of the respondent's working hours were 6–8; however, 41.50% had no fixed working hours. Using the WHO classification, samples were divided into four BMI groups and then the overweight and obese was merged. This study reports n = 28 (7.0%) are in the underweight group, n = 294 (73.50%) are in the normal group, and n = 78 (19.50%) in the overweight and obese groups, respectively.

**Table 1:** Sociodemographic characteristics of the respondents (n = 400).

Variables	Frequency	Percentage
Place of residence		
Dhaka	202	50.50
Chattogram	116	29.00
Gazipur	82	20.50
Gender		
Male	274	68.50
Female	126	31.50
Age group		
18–30 years	267	66.75
31–40 years	61	15.25
41–50 years	45	11.25
51–60 years	27	6.75
Current marital status		
Married	194	51.50
Single	206	48.50
Family type		
Nuclear	272	68.00
Joint family	96	24.00
Life apart from family	32	8.00
Education		
HSC and below	205	51.25
Graduation and above	195	48.75
Field of study		
Biological science	120	30.00
Other than biological science	280	70.00
Occupation		
Service	137	34.25
Business	61	15.25
Others (Homemaker, unemployed, student)	202	50.50
Working hours		
6–8 h	135	33.75
10 h and more	99	24.75
Not fixed	166	41.50
Income		
< 30,000	184	46.00
30,000–60,000	147	36.75
60,000–90,000	4444 44	11.00
> 90,000	25	6.25
BMI categories		
Underweight	28	7.00
Healthy weight	294	73.50
Overweight and obese	78	19.50

*HSC* Higher Secondary School Certificate (12 grade), income describes in BDT (Bangladeshi Taka).

### Perceived barriers to physical activity

**Figure 1:**
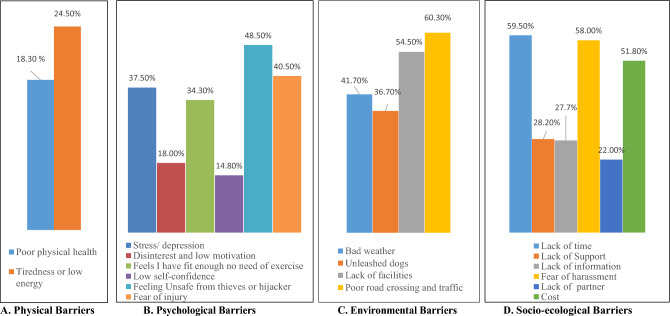
The agreements and non-agreements for perceived barriers to physical activity.

The agreements and non-agreements for perceived barriers to physical activity are summarized in Table 2 and in Fig. 1. About18.30% of respondents reported poor physical condition as an agreement, and 24.50% had tiredness. Approximately 37.50% of respondents agreed with depression/stress as a barrier, and 34.30% believed they were fit enough as they did not need physical exercise. About 48.50% of the participants felt unsafe from thieves or hijackers when they intended to have physical activity. A total of 40.50% had a fear of injury during physical activity. Bad weather, unleashed dogs, and lack of facilities (walking or physical activity-related) were considered barriers by 41.70%, 36.70%, and 54.50% of the study participants, respectively. The Most agreed barrier (60.30%) of physical activity was poor road traffic and construction work besides the walkway. As socio-ecological barriers, 59.50% had a lack of time, 58.00% feared harassment, and the cost was reported by 51.80% of respondents as an agreement of perceived barriers to physical activity.

**Table 2:** Perceived barriers to physical activity among adults living in three major cities of Bangladesh (n = 400).

Perceived barriers items	Agreement (%)	Non-agreement (%)
Physical barrier		
Poor physical health	18.30	81.70
Tiredness or low energy	24.50	75.50
Psychological barriers		
Stress/ depression	37.50	62.50
Disinterest and low motivation	18.00	82.00
Feels I have fit enough no need of exercise	34.30	65.70
Low self-confidence	14.80	85.20
Feeling unsafe from thieves or hijackers	48.50	51.50
Fear of injury	40.50	59.50
Environmental barriers		
Bad weather	41.70	58.30
Unleashed dogs	36.70	63.30
Lack of facilities (like pavements, parks, and walkways)	54.50	45.50
Poor road crossing and traffic and construction work besides walking way	60.30	39.70
Socio-ecological barriers		
Lack of time	59.50	40.50
Lack of Support	28.20	71.80
Lack of information	27.80	72.20
Fear of harassment (Especially for females)	58.00	41.00
Lack of partner	22.00	78.00
Cost	51.80	48.30

### Mental health status

**Figure 2:**
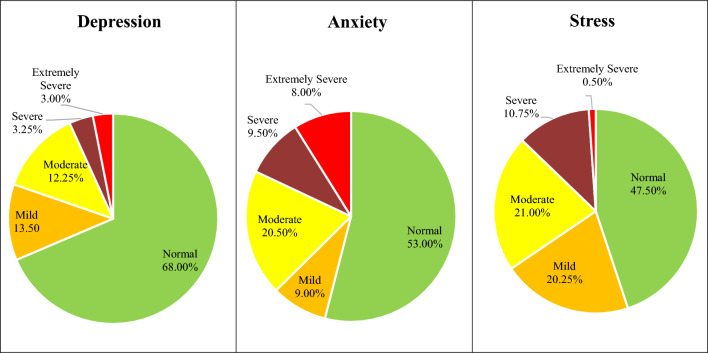
Prevalence of depression, anxiety, and stress among the study participants.

Figure 2 illustrates the mental health status of the participants obtained by the DASS-21 score. Among the 400 participants, the score reported mild to extremely severe levels of depression (32%), anxiety (47%), and stress (42.50%).

### Multivariable quantile regression analysis

A multivariable quantile regression was fitted on each of the 10th, 25th, 50th, 75th, and 90th quantiles of the scores for perceived barriers to physical activity to show a complete picture of the association between the explanatory variables and physical activity barrier scores. Before the multivariate modeling, we performed the bivariate analysis, presented in a supplementary file (Table S1). The outputs for the quantile regression, and the OLS regression models are presented in Table 3. The model estimate suggests that gender is significantly associated with higher perceived barriers to physical activity scores at the 10th quantile (95% CI 2.78–10.23), 25th quantile (95% CI 5.39–12.13), 50th quantile (95% CI 3.63–10.83) and 90th quantile (95% CI 2.46–11.43), after adjusting for other covariates. However, marital status was statistically significant in only the 25th quartile (95% CI − 8.51 to − 0.70). Respondents who lived apart from home tended to have higher scores on perceived barriers to physical activity compared to the nuclear family members at the 10th, 25th, and 50th quantiles. The businessman’s median score for the perceived barriers to physical activity was observed to be 5.57 points higher than those who were service holders (95% CI 1.11–10.03). The observed value for the physical activity barrier score at the 75th quantile also showed statistical significance for the business professional.

**Table 3:** Multivariate analysis on perceived barriers to physical activity (n = 400).

Predictors	Linear regression mean	10th quantile	25th quantile	50th quantile	75th quantile	90th quantile
Age	0.12 (− 0.02 to 0.26)	− 0.09 (− 0.37 to 0.19)	0.11 (− 0.05 to 0.28)	0.12 (− 0.05 to 0.31)	0.06 (− 0.14 to 0.26)	**0.16 (0.02 to 0.31)**
Gender						
Male (Ref)						
Female	7.49 **(4.90 to 10.08)**	6.51 **(2.78 to 10.23)**	8.76 **(5.39 to 12.13)**	7.23 **(3.63 to 10.83)**	6.94 **(2.46 to 11.43)**	4.46 (− 0.53 − 9.45)
Marital status						
Unmarried (ref)						
Married	− 2.66 (− 5.69 to 0.35)	− 2.55 (− 7.29 to 2.18)	− 4.60 **(− 8.51 to − 0.70)**	− 2.22 (− 5.66 to 1.21)	− 1.94 (− 6.73 to 2.84)	− 0.42 (− 6.04 to 5.19)
Family type						
Nuclear (ref)						
Joint	1.73 (− 0.82 to 4.29)	1.21 (− 2.75 to 5.18)	1.84 (− 1.85 to 5.54)	1.21 (− 3.92 to 6.34)	2.55 (− 0.97 to 6.08)	3.57 (− 2.04 to 9.19)
Live apart home	6.87 **(2.80 to 10.95)**	**7.39 (1.10 to 13.68)**	8.07 **(3.82 to 12.31**)	**7.36 (2.48 to 12.23)**	4.67 (− 1.40 to 10.75)	4.71 (− 3.69 to 13.12)
Occupation						
Service (ref)						
Business	3.82 (− 0.02 to 7.67)	− 0.52 (− 6.81 to 5.76)	5.10 (− 2.47 to 12.67)	**5.57 (1.11 to 10.03)**	**6.45 (1.01 to 12.07)**	2.75 (− 5.29 to 10.80)
Others	0.67 (− 2.49 to 3.38)	0.06 (− 3.74 to 3.88)	1.82 (− 2.01 to 5.67)	0.15 (− 3.15 to 3.46)	0.97 (− 4.41 to 6.36)	0.44 (− 6.70 to 7.60)
Work duration						
6 h	− 3.25 (− .98 to 2.47)	− 3.39 (− 10.83 to 4.04)	− 6.65 (− 13.81 to 0.50)	− 10.08(− 22.0 to1.86)	0.75 (− 13.91 to 15.41)	10.64 (− 10.37 to 31.67)
8 h. (ref)						
10 h. ore more	2.49 (− 0.84 to 5.83)	4.99 **(0.06 to 09.93)**	3.94 (− 1.42 to 9.32)	2.06 (− 1.41 to 5.54)	0.29 (− 5.19 to 5.78)	− 0.83 (− 8.98 to 7.30)
Not fixed	− 1.43 (− 4.85 to 1.99)	− 1.17 (− 6.14 to 3.79)	− 3.15 (− 9.46 to 3.16)	− 2.54 (− 6.16 to 1.07)	− 1.39 (− 9.47 to 6.68)	− 0.83 (− 9.98 to 8.31)
Income						
< 30,000	5.56** (0.81 to 10.32)**	2.56 (− 4.54 to 9.67)	5.87 **(0.31 to 11.43)**	4.62 (− 1.05 to 10.29)	8.27 **(1.27 to 15.28)**	12.42 **(7.82 to 17.0)**
30,000–60,000	2.51 (− 2.13 to 7.17)	− 0.22 (− 8.79 to 8.35)	2.88 (− 2.34 to 8.12)	2.10 (− 2.86 to 7.06)	6.53 (− 1.21 to 14.27)	9.35 **(2.84 to 15.87)**
60,001–90,000	3.74 (− 1.63 to 9.12)	− 1.56 (− 9.71 to 6.59)	2.37 (− 3.81 to 8.56)	4.71 (− 4.96 to 14.39)	6.36 (− 2.42 to 15.15)	8.49 (− 6.14 to 23.13)
> 90,000 (ref)						
BMI	0.48** (0.08 to 0.87)**	0.22 (− 0.48 to 0.92)	0.61 **(0.01 to 1.21)**	0.40 (− 0.09 to 0.90)	0.29 (− 0.20 to 0.79)	0.10 (− 0.49 to 0.70)
Depression score	− 0.004 (− 0.20 to 0.19)	− 0.19 (− 0.52 to 0.s12)	− 0.19 (− 0.44 to 0.05)	− 0.14 (− 0.31 to 0.02)	0.05 (− 0.25 to 0.37)	0.22 (− 0.29 to 0.74)
Anxiety score	0.37 **(0.17 to 0.57)**	0.40 **(0.20 to 0.60)**	**0.33 (0.06 to 0.59)**	0.45** (0.13 to 0.78)**	0.49 **(0.06 to 0.92)**	0.43 (− 0.07 to 0.94)
Stress score	0.36 **( 0.19 to 0.51)**	0.15 (− 0.09 to 0.40)	**0.32 (0.09 to 0.56)**	0.41 **(0.17 to 0.65)**	0.41 **(0.17 to 0.66)**	0.51 **(0.26 to 0.75)**

Significant values are in **bold**.

Model estimates and 95% CI are displayed for each predictors of perceived barriers to physical activity scores. P values < 0.05 were found to be statistically significant.

*Ref:* Reference category, In this table we presented only those explanatory variables which was significance at least one of the quantile regression analysis.

Looking at the working hours, the 10th quantile was found to be a statistically significant predictor for the higher perceived barriers physical activity scores for the participants who had 10 h or more long work schedule had 4.99 points higher score than those who did 6-h jobs (95% CI 0.06–09.93), after adjusting for other explanatory variables. Lowest-income (< 30,000) groups’ physical activity score was statistically significant at the 25th (95% CI 0.31–11.43), 75th (95% CI 1.27–15.28), and 90th quantile (95% CI 7.82–17.0) compared to the highest-income (> 90,000 BDT) group. The estimated 25th quantile of perceived barriers to physical activity increased by 0.61 points (95% CI 0.01–1.21) if the BMI increased by one point. Anxiety was significantly associated with the perceived barriers to physical activity in every five quantiles except the 90th quantile. Stress was also statistically significantly associate with perceived barrier scores at the 25th, 50th, 75th, and 90th quantiles.

## Discussion

To the authors' knowledge, no other studies examined the perceived barriers to physical activity and its association with mental health status among Bangladeshi adults. This study is the first to identify the perceived barrier to physical activity among the 18 to 60 age group. Moreover, barriers were assessed in four specific domains not previously considered in Bangladesh. In addition, this study identified a few other factors that act as a barrier in the context of Bangladesh, such as, construction work next to the road, unleashed dogs in the streets and parks, mental distress, fear of harassment, etc. Additionally, we incorporated a few other dimensions to the barrier; for example, we tried to capture emotional barrier via family type (those who lived apart from family faced more obstacles than those who lived with their family). Finally, we also included the mental health aspect in this study and showed how mental health might be a barrier to physical activity.

Five main barriers were perceived by the study participants, namely, poor traffic system, lack of facilities, feeling unsafe, lack of time, and fear of harassment. Tiredness or low energy was perceived as the main reported barrier in the physical domain. “Feel I am fit enough, so no need to do exercise”, “Low self-confidence”, and “Feeling unsafe from thief and hijacker” was reported as psychological barriers to physical activity. These findings are align with previous research from other countries^38,39^.

This study claimed bad weather, unleashed dogs, lack of facilities, and poor road traffic systems as environmental barriers aligned with previous research^38,40^. In recent years, climate change and bad weather have been experienced in Bangladesh, which harms city life and livelihoods almost every year due to population density, rural–urban migration, unplanned urbanization, and a lack of public utilities^41^. Research conducted in India explored the significant barriers to regular physical exercise: poor infrastructure maintenance, lack of cleanliness, lack of outdoor and indoor spaces nearest to the house, and unfavorable weather conditions^42^. Due to the proximity in terms of environment and development, Bangladesh and India face similar environmental challenges; consequently, the people's perceptions in both countries are similar^43,44^.

The most frequently reported environmental barrier to physical activity in this study was poor road traffic and construction work besides the roads, as stated by more than 60% of the respondents. Many city footpaths are still inaccessible, primarily because of the dumping of construction materials, illegal parking of cars, pop-up stores, and street vendors^45^. Previous studies conducted in Bangladesh have also drawn attention to the fact that the notable environmental barriers were feeling unsafe in walking in their neighborhood, heavy traffic, poor footpath conditions^17^. However, this finding was consistent with our assessment, and we observed that young adults were not the only ones who face these barriers, working people in the major cities of Bangladesh experienced similar barriers.

Lack of time was a considerable perceived barrier to physical activity in this study. Several studies also aligned with this perception. In a study by Koh et al. the lack of time (65.3%) was reported as the most frequently stated perceived barrier^22^. Uijtdewilligen et al. revealed the top three reasons for not visiting parks to do physical activity were being too busy with their work or studies, being too tired or staying at home, and being concerned about the weather^46^. The explanation could be that engagement of the study participants in various activities or lack of motivation may avert them from setting aside time to engage in physical activities. According to the findings, more than half of the study participants feared harassment during physical activity and outdoor exercise. Safety concerns concerning physical activity have been studied extensively concerning different realms of physical activity. Harassment is a harsh reality in Bangladesh, especially for women^47^. Women’s perceived barriers to physical activity were more prominent than men in the current study, even though most participants were male. A recent study reported that males showed empathy about the harassment and violence against women^48^. The reason for more male participants in our study is that females are more involved in household activities and the outdoor responsibilities are considered the males^49^. Moreover, female participation in tertiary education, employment, and business ventures is still lower males^50^. Given that data were primarily collected from central business district and shopping malls, our study had more male participants than female participants. Additionally, regarding the respondents’ participation in this study, males were more responsive than females. Similar findings were observed in previous studies, where women faced more barriers to physical activity than men^47,48^. In this study, respondents who lived away from their families perceived more obstacles to physical activity, suggesting that family support plays a crucial role in active behavior. Individuals who reside away from their family may feel lonely, and their motivation and encouragement towards engaging in physical activity may remain low compared to the ones who lives with their family or friends. Additionally, the literature suggests that the family environment is conducive to healthy physical and mental development^51^. A Brazilian research supports this finding, as they found the family promotes transformations in behavioral patterns among family members and perceived them adopting healthy habits to become physically active^52^. Since PA-related habits, values, and beliefs are learned within the family environment, the research states that family-based approaches have great potential to promote and support youth PA within the family and home setting^53^.

According to the current research, the participants who worked in business were likely to be less active than the service holders. This could be because of work-life pressure, a busy lifestyle, or the fact that business people may not be surrounded by people who want to be active, like coworkers, family, or friends, who can encourage them to be involved at work or outside of work. The current results align with other research, which is that the workplace environment and how coworkers act may be a barrier to physical activity^52,54^. This study also revealed that an individual who works less than six hours had a significantly lower physical activity barriers score compared to those who had 10 h or more than 10 h work schedule. These results are also supported by previous research^55^. People who work long hours are more likely to be physically inactive than those who work shorter hours, mainly because they have less time and are tired to exercise outside of work^45^. Less monthly income was also reported as an important barrier to physical activity. People who earned less than 30,000 BDT per month had higher physical activity barrier scores than those who earned more than 90,000 BDT. Prior studies have suggested an association between socioeconomic status (SES) and participation in physical activity, with those with higher SES typically being more physically active than those with lower SES^56^. Financially constrained people cannot manage time to do physical exercise apart from their professional work^57^. On the contrary, evidence reveled opposite findings to our result. Stalsberg et al. conducted a review that revealed inconclusive evidence that the high- or low-SES group was more physically active; their study also suggested PA comparisons across SES groups should account for total PA and leisure time PA^58^.

The current study found that most participants experienced various degrees of mental distress, which was associated with increased perceived barriers to physical activity. The mental health care in Bangladesh is suboptimal because it doesn't receive adequate fund allocation, insufficient public mental health facilities, insufficient qualified mental health experts, and lack of well-run mental health policies^59^. Prior research also aligned with our findings, as individuals with mental disorders may be less motivated to engage in physical activity^23,60^. Hasan et al. found mental diseases were common and usually more severe across all age groups in Bangladesh^59^. In contrast, a few pieces of existing literature have presented counter-evidence suggesting that individuals with mental illness may face greater challenges in making health behavior changes towards continues regular physical activity than the general population^61,62^.

There are several strengths of this study. First, people’s perceptions of barriers to physical activity and their association to mental health status were assessed among Bangladeshi nationals’, which is the first study of this nature. Another strength was using a robust statistical technique, quantile regression, to determine the association between barriers to physical activity and other covariates. This study had a reasonably large sample size. This study also has a few shortcomings. Given it was a cross-sectional design, this study cannot establish a causal relation between the perceived barriers to physical activity and other covariates. Another limitation was that the scale used to assess perceived barriers to PA in this study had not been previously validated in Bangladesh. Finally, this study collected samples from three major cities of Bangladesh, which may not represent the whole population of Bangladesh. Hence the results obtained from this study may not be generalizable.

The findings of this research have several practical implications for policymakers, healthcare professionals, public health organizations, and stakeholders. First, the study identified the most common barriers to physical activity among Bangladeshi adults, including environmental, physical, and psychological factors. Therefore, public health programs should address these barriers through targeted interventions, such as promoting workplace wellness programs, community-based physical activity programs, and accessible and affordable exercise facilities. Additionally, policy initiatives may be implemented to increase access to affordable and convenient exercise facilities for the targeted groups, such as public parks and recreation centers. Healthcare professionals may consider the associated factors to screen patients for relevant mental health issues and provide appropriate interventions, such as counseling and therapy, to promote physical activity and to improve mental health outcomes. Future recommendations for research could include examining the effectiveness of these targeted interventions in addressing the perceived barriers to physical activity and identifying additional factors that may influence physical activity behavior among adults. Furthermore, exploring the impact of technology-based interventions and virtual physical activity programs could be an exciting avenue for future research.

## Conclusions

Perceptions of barriers to physical activity varied according to gender, marital status, family type, occupation, working hours, income, and mental health status. Ensuring a safe environment, facilitating accessibility and availability of low-cost exercise facilities, improving road and traffic conditions, and providing appropriate motivation and counseling to reduce stress, anxiety, and depression may help to mitigate an individual’s physical activity barriers. Findings from this research will aid people as well as healthcare providers, policymakers, and stockholders in implementing preventative measures to encourage and facilitate physical activities.

## Methods

### Study design, sampling and participants’

**Figure 3:**
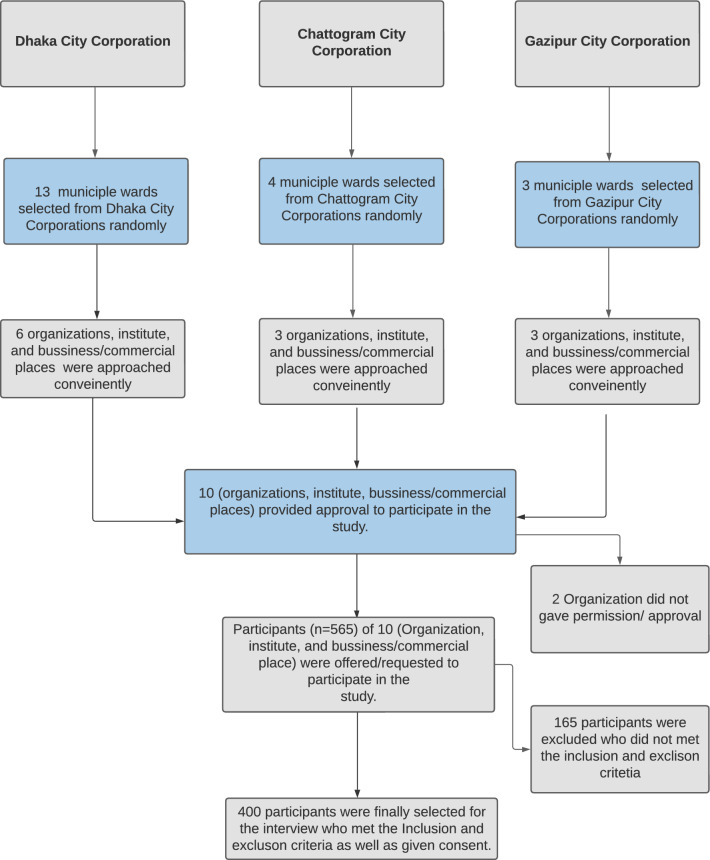
Flow chart of the sample selection criterion.

This is a cross-sectional study was conducted in three city corporations (Dhaka, Chattogram, and Gazipur) in Bangladesh from January to June 2022. Participants of both sexes, between the ages of 18 and 60 years, living in different areas of Dhaka, Chattogram, and Gazipur city corporations, were included in this study. However, Participants who were injured, in a rehabilitation stage, or unwilling to participate were excluded from the study. The sample size was calculated using the formula of Cochran’s (n = (z^2 * p *(1-p))/e^2^). With 5% margin of error (e), considering the physical activity barrier prevalence (p = 63%) as reported in a previous study in India^42^, and the standard normal variate of 1.96 (z), the required sample size was estimated as 358. However, the study team reached a higher sample of 565. One hundred and sixty-five participants were excluded from the study based on the inclusion and exclusion criteria. Excluded participants were either outside of the age range of this study, did not live in the city where we were conducting the study, etc. Finally, 400 respondents were included in this study for analysis. This study employed a multistage sampling technique to determine the study participants. The details of the sampling procedure have been presented in Fig. 3. In the first stage, 20 wards (sub-division of city corporations) from three city corporations were randomly selected. Study participants were conveniently selected from each municipal ward at the final stage. Selected wards are presented in a map and added as a supplementary document (Fig. S1). Approximately 50% of the samples were selected from the Dhaka city corporation and the remaining samples were collected from the other two city corporations. The reasoning for having such split is: the total population of Dhaka city corporation is 22,500,000^63,64^; conversely, the sum of the people in Gazipur and Chattogram City Corporations is 1,05,0000, nearly half of the Dhaka city corporation's population^65,66^.

### Data collection procedure

Five trained surveyors/data collectors were used in the data collection process who, in turn, was placed in the central business district, shopping malls, and in the educational institutions to capture diverse study population. Participants are approached and a brief description of the study was conveyed. Once the participants provided consent to participate in this study, sample data were then collected. Data were collected through face-to-face interviews with a semi-structured and pretested questionnaire. We first formed all of the questionnaires in English, including the questions about barriers to physical activity. Then, a professional Bengali translator translated them into Bengali. The participants may choose to use a Bengali or an English questionnaire; however, most responded in English. We included the English version of the questionnaire as a supplementary document. The questionnaire included socio-demographics, perceived barriers to physical activity, and mental health-related questions. A draft version of the questionnaire in a small sample from Dhaka City has been piloted to evaluate feasibility and acceptability.

### Measurements

#### Perceived barriers to physical activity

Perceived barriers to physical activity-related questionnaire were designed based on previously published studies^15,67^. Unfortunately, we could not find any validated questionnaire for the age group we studied. Therefore, we borrowed seemingly appropriate questions from those literatures, although they were applied to different population groups. Our rationale for following this strategy was based on previous literature. Combining questions from those studies, AlQuaiz et al. conducted a research study to measure the obstacles to physical activity among participants aged between 30 and 75 years^68^. Phidelia et al. assessed physical activity barriers among nurse trainees^69^. Moreover, the reliability value was assessed as 84% when we did the pilot study. Participants were asked 18 questions to measure perceived barriers to physical activity. A 5-point Likert scale ranging from “not a problem” to “a significant problem” was used to measure the barriers score. The scale has four domains: physical barriers, psychological barriers, environmental barriers, and social-surrounding barriers. The responses were classified into two groups: those in agreement (answers 4 and 5 options on the Likert scale) and those in disagreement (answers 1, 2, and 3). The response of the barriers scale was also cumulated to overall PA score. The reliability value of the perceived barrier to physical activity questionnaire was 0.84.

#### Depression, anxiety, and stress (DASS-21)

The DASS-21 scale is a valid and reliable scale for measuring psychological health. The Scale reliability coefficient (Cronbach’s alpha) for Depression, Anxiety and Stress was 0.85. DASS-21 is a condensed version of the 42-item DASS scale, which consists of the depression, anxiety, and stress subscales^70^. There are seven items in each of the three DASS-21 sub-scales. This well-known and widely used DASS-21 scale has been translated and validated in Bengali^71^. On a four-point Likert scale which ranged from 0 (never) to 3 (almost always), respondents were questioned about their level of mental distress over the previous four weeks. Individual depression, anxiety, and stress scores were calculated by summing the scores for their respective 7 items. The final score for each of the 3 dimensions was then multiplied by two to obtain a score between 0 and 42^72,73^. Individual scores for each of these 3 subscales were then categorized into five severity categories as: normal, mild, moderate, severe, and extremely severe^74^. The following Table 4 describes what score puts an individual into each of these five categories.

**Table 4:** Recommended cut-off for Depression Anxiety and Stress scale^75^.

Severity	Depression	Anxiety	Stress
Normal	0–9	0–7	0–14
Mild	10–13	8–9	15–18
Moderate	14–20	10–14	19–25
Severe	21–27	15–19	26–33
Extremely severe	28 +	20 +	34 +

### Sociodemographic variables

Participants also filled out questions to provide their sociodemographic data about their age, gender, height, weight, marital status, type of family, education, the field of study, occupation, gross monthly household income, and daily working hours. Individuals were subclasses into four different groups based on their age. Participants' self-reported height and weight were used to determine their body mass index (BMI). The BMI was then categorized based on the WHO's major cut-off points: normal range (18.50–24.99 kg/m^2^), underweight (18.50 kg/m^2^), the overweight (25.00 kg/m^2^), and obese (> 29.9 kg/m^2^)^76^. Later, overweight and the obese categories were combined into one group. Monthly gross household income was used to represent socioeconomic status and put into four groups: <  = 30 000 Bangladeshi currency (BDT), 30 001–60 000 BDT, 60 001–90 000 BDT, and > 90 000 BDT.

### Statistical analysis

The frequencies and percentages were used to narrate the baseline characteristics of the respondents. The Shapiro–Wilk test and a histogram checked the normality of outcome variable. Wilcoxon rank sum test and Kruskal–Wallis, and Spearman’s rank correlation test were applied to assess the bivariate analysis as we found our outcome measurements as non-normally distributed. We performed the bivariate analysisto examine the association between the perceived barriers to physical activity and other covariates. In the multivariate modeling, we adjusted for age, gender, marital status, family type, education, the field of study, occupation, work duration, Income, BMI, depression score, anxiety score, and stress score. We opt to include all the explanatory variables in the regression model irrespective of their significance in the bivariate relationship. According to Lo et al., including only statistically significant variables resulted from bivariate analysis is risky because some variables that are not significant in a bivariate analysis might become so in a multivariate analysis^77^.

Quantile regression analyses were used to determine how each covariate affected the perceived physical activity barrier scores on average. A linear regression analysis was also accompanied for comparison purposes. The ordinary least squares regression generates model-based mean estimates only, whereas the Quantile regression generates estimates of a given quantile. Therefore, the tail of the distribution for the perceived barrier scores may also be modeled using quantile regression to examine explanatory variables' effects on extreme quantiles. Five quantiles were used: the 10th, 25th, 50th, 75th, and 90th. The hypothesis tests were two-sided, and the p-values less than 0.05 were considered significant. The STATA (V16 StataCorp LP, TX, USA) software was used for the analyses.

### Ethical approval

The North South University Ethics Review Committee provided ethical approval for this research protocol. Before participation, the study’s objectives and nature were expressed to and explained to the respondents. An informed consent was obtained from all the participants. All the people who took part in the study signed a written statement saying they understood the study and agreed to take part. The Declaration of Helsinki's principles were followed in this study.

## Supplementary Information

Supplementary Figure S1.

Supplementary Table S2.

## Data Availability

The data underlying this article will be provided by the corresponding author on a reasonable request.

## Abbreviations

BDT: Bangladeshi Taka (Currency)
BMI: Body mass index
DASS-21: Depression anxiety and stress scale (21 items)
LMICs: Low and middle income countries
NCDs: Non communicable diseases
PA: Physical activity
SES: Socioeconomic status
WHO: World health organizations
