# Supplementary material for: Perceived barriers to maintain physical activity and its association to mental health status of Bangladeshi adults: a quantile regression approach
Source: Sci Rep. 2023 Jun 2;13:8993. doi: 10.1038/s41598-023-36299-7 (PMC10238517; doi:10.1038/s41598-023-36299-7)
Supplement: Supplementary file 1 — Supplementary Figure S1. [file 41598_2023_36299_MOESM1_ESM.docx]

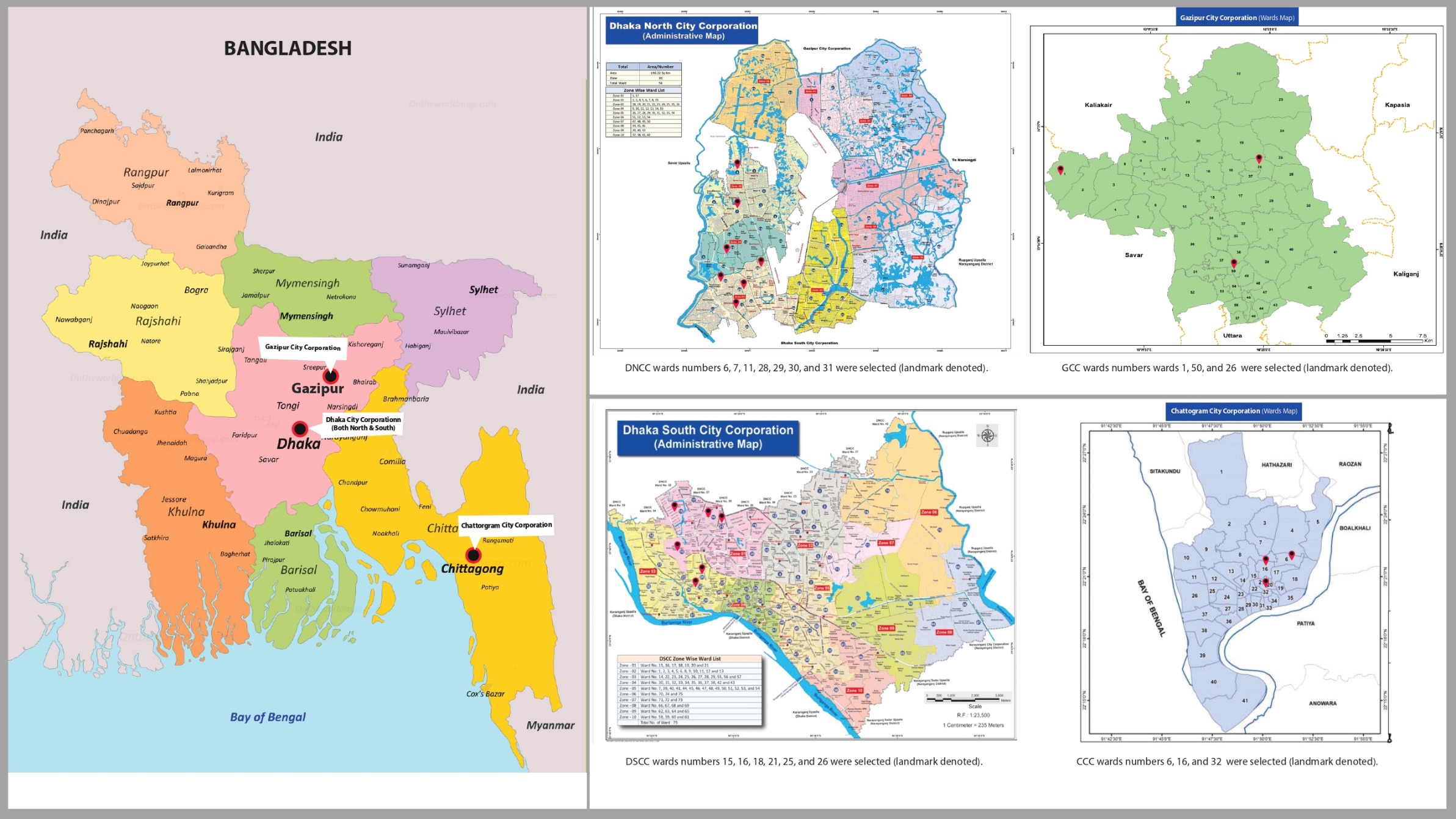
**Figure S1.** The map of the three city corporations of Bangladesh and selected study sites. The map was created using ArcGIS Pro version 2.0 (https://www.esri.com/en-us/arcgis/products/arcgis-pro/overview)
