# Supplementary material for: Perceived barriers to maintain physical activity and its association to mental health status of Bangladeshi adults: a quantile regression approach
Source: Sci Rep. 2023 Jun 2;13:8993. doi: 10.1038/s41598-023-36299-7 (PMC10238517; doi:10.1038/s41598-023-36299-7)
Supplement: Supplementary file 2 — Supplementary Table S2. [file 41598_2023_36299_MOESM2_ESM.docx]

**Table S1: Bivariate analysis between the perceived barriers to physical activity score and the explanatory variables**

| **Independent Variables** | **Perceived Barriers to Physical Activity Score** | **Perceived Barriers to Physical Activity Score** |
| --- | --- | --- |
|  | **P-value (Parametric tests)** | **P-value(Non parametric test)** |
| **Gender^!^** | **<0.001** | **<0.001** |
| Male |  |  |
| Female |  |  |
| **Age Group** |  |  |
| 18-30 Years | 0.549 | 0.294 |
| 31-40 Years |  |  |
| 41-50 Years |  |  |
| 51-60 Years |  |  |
| **Current marital status^!^** |  |  |
| Married | 0.942 | 0.859 |
| Single |  |  |
| **Family type** |  |  |
| Nuclear | 0.349 | 0.083 |
| Joint family |  |  |
| Life apart from family |  |  |
| **Education^!^** |  |  |
| HSC and below | 0.566 | 0.276 |
| Graduation and above |  |  |
| **Field of Study ^!^** |  |  |
| Biological science | 0.072 | **0.031** |
| Other than biological science |  |  |
| **Occupation** |  |  |
| Service | 0.473 | 0.144 |
| Business |  |  |
| Others(Homemaker, unemployed, student) |  |  |
| **Working hours** | **0.030** | 0.060 |
| 6-8 Hour |  |  |
| 10 Hours and more |  |  |
| Not fixed |  |  |
| **Income** | 0.409 |  |
| <30000 |  | 0.087 |
| 30000-60000 |  |  |
| 60000-90000 |  |  |
| >90000 |  |  |
| **BMI** | 0.0407 | 0.907 |
| **Depression Score** | 0.2469 | **0.039** |
| **Anxiety Score** | 0.3382 | **<0.001** |
| **Stress Score** | 0.3580 | **<0.001** |

*p-value <0.05 was found to be statistically significant. p values were obtained by Wilcoxon rank sum test^!^ Kruskal Wallis test, and Spearman rank sum test for non-parametric tests. Where Independent sample t-test, one-way ANOVA and Pearson correlation test were applied for parametric test.*
